# The ciliary Frizzled-like receptor Tmem67 regulates canonical Wnt/β-catenin signalling in the developing cerebellum via Hoxb5

**DOI:** 10.1038/s41598-019-41940-5

**Published:** 2019-04-01

**Authors:** Zakia A. Abdelhamed, Dina I. Abdelmottaleb, Mohammed E. El-Asrag, Subaashini Natarajan, Gabrielle Wheway, Chris F. Inglehearn, Carmel Toomes, Colin A. Johnson

**Affiliations:** 1 Divison of Molecular Medicine, Leeds Institute of Medical Research, University of Leeds, LS9 7TF Leeds, UK; 2 Department of Zoology, Faculty of Science, Benha University, Benha, Egypt; 3 Human Development and Health, Faculty of Medicine, University of Southampton, SO16 6YD Southampton, UK; 4 Present Address: Division of Human Genetics, Cincinnati Children’s Hospital, 3333 Burnet Avenue, Cincinnati, OH 45229-3039 USA

## Abstract

Primary cilia defects result in a group of related pleiotropic malformation syndromes known as ciliopathies, often characterised by cerebellar developmental and foliation defects. Here, we describe the cerebellar anatomical and signalling defects in the *Tmem67*^*tm1*(*Dgen*)/*H*^ knockout mouse. At mid-gestation, *Tmem67* mutant cerebella were hypoplastic and had aberrantly high canonical Wnt/β-catenin signalling, proliferation and apoptosis. Later in development, mutant cerebellar hemispheres had severe foliation defects and inferior lobe malformation, characterized by immature Purkinje cells (PCs). Early postnatal *Tmem67* mutant cerebellum had disrupted ciliogenesis and reduced responsiveness to Shh signalling. Transcriptome profiling of *Tmem67* mutant cerebella identified ectopic increased expression of homeobox-type transcription factors (*Hoxa5*, *Hoxa4*, *Hoxb5* and *Hoxd3*), normally required for early rostral hindbrain patterning. HOXB5 protein levels were increased in the inferior lobe, and increased canonical Wnt signalling, following loss of TMEM67, was dependent on HOXB5. HOXB5 occupancy at the β-catenin promoter was significantly increased by activation of canonical Wnt signalling in *Tmem67*^−/−^ mutant cerebellar neurones, suggesting that increased canonical Wnt signalling following mutation or loss of TMEM67 was directly dependent on HOXB5. Our results link dysregulated expression of Hox group genes with ciliary Wnt signalling defects in the developing cerebellum, providing new mechanistic insights into ciliopathy cerebellar hypoplasia phenotypes.

## Introduction

Primary cilia are microtubule-based organelles that sense and transduce extracellular signals on many cell types during the G_1_/G_0_ phase of the cell cycle^1–3^, and it is now well-established that cilia mediate key pathways of embryonic development, including Wnt and Shh signalling^4–6^. An important group of inherited developmental disorders, the ciliopathies, are caused by mutations in genes encoding proteins that are components of the primary cilium and basal body^7,8^. The cerebellum is the main component of the posterior fossa and it is the brain structure most commonly affected in ciliopathies. Cerebellar developmental and foliation defects are a specific finding in a number of ciliopathies that include Joubert syndrome (JBTS)^9–12^, Meckel-Gruber syndrome (MKS)^13–15^, Bardet-Biedl syndrome (BBS)^16^, and oro-facial-digital (OFD) syndrome^17,18^. The presence of cerebellar abnormalities in such a broad range of distinct, although highly related, pleiotropic ciliary disorders suggests that cilia have critical functions during cerebellar development. Both granule cell progenitors (GCP), forming the external granular layer (EGL) of cells^19,20^, and the Purkinje cells (PCs)^21^ of the developing cerebellum are ciliated. Conditional ablation of the *Kif3a* and *Ift88* genes, which are both essential for cilia formation, in the GCP layer of the mouse cerebellum resulted in severe hypoplasia of the cerebellum and pronounced foliation defects, suggesting that defects in granule cell proliferation are a cause of cerebellar abnormalities in ciliopathies^22^. In JBTS patients, the abundance of the GCP cells is markedly diminished and this has also been attributed to the diminished proliferation of these cells^23^.

Mutations in the *TMEM67* gene are a major cause of human MKS and JBTS^14,15^, both presenting with severe cerebellar abnormalities. *TMEM67* encodes the Frizzled-like Wnt receptor TMEM67 (also known as meckelin) that we have recently shown functionally interacts with the ligand Wnt5a^24^. Cerebellar defects have not been reported in previously described *Tmem67* rodent models such as *Tmem67*^*tm1*(*Dgen*)/*H*^ ref.^25^, the *bpck* mouse^26^ and the *Wpk* rat^27^. However, our characterization of the *Tmem67*^*tm1*(*Dgen*)/*H*^ knock-out model (hereafter designated *Tmem67*^−/−^) showed that a variable MKS-like phenotype (neural tube defects, occipital meningocele, midbrain-hind brain exencephaly and frontal encephalocele) at embryonic day E11.5 of gestation was associated with abberently increased Wnt/β-catenin signalling both *in vitro* and *in vivo*^28^. *Tmem67*^−/−^ mutant phenotypes in later gestation (after E15.5) revealed phenotypes compatible with JBTS (cerebellar vermis hypoplasia or aplasia, deep interpeduncular fossa and posterior fossa defects). However, it is unclear what role the TMEM67 receptor has in mediating ciliary signalling pathways, specifically the Shh and Wnt pathways, during later cerebellar development^28^.

To address this, the present study describes the anatomical and signalling defects in the developing *Tmem67*^−/−^ mutant cerebellum during later gestation (after E15.5). We demonstrate impaired development of the cerebellum and the pronounced foliation defects in all *Tmem67*^−/−^ animals during late gestation. These were concomitant with reduced responsiveness to ciliary-dependent Shh signalling and perturbation of downstream gene expression patterns that are normally associated with rostral hindbrain patterning. These results extend previous observations in human ciliopathy patients^29^, highlight the suitability of *Tmem67*^*tm1*(*Dgen*)/*H*^ as a model of human ciliopathies, and suggest new pathways that are regulated by primary ciliary signalling during cerebellar development and maturation.

## Materials and Methods

### Animals

The animal studies described in this paper were carried out under the guidance issued by the Medical Research Council in *Responsibility in the Use of Animals for Medical Research* (July 1993) in accordance with UK Home Office regulations under the Project Licence no. PPL40/3349. All experimental protocols were approved by the University of Leeds Animal Welfare and Ethical Review Committee. C57BL/6 J;129P2-*Tmem67*^*tm1*(*Dgen*)/*H*^ heterozygous knock-out mice were derived from a line generated by Deltagen Inc. and made available from MRC Harwell through the European Mutant Mouse Archive (EMMA) repository (see https://www.infrafrontier.eu/ strain number EM:02370). The *tm1* reporter-tagged deletion allele was created by targeted *β-Gal-neo* (“*geo*”) construct insertion downstream of exon one of the *Tmem67* gene. Genotyping was carried out by PCR on DNA extracted from tail tips or the yolk sac of the E11.5 embryos, or tail or ear biopsies of older embryos and adult mice, respectively. Animals were back-crossed onto the C57BL/6J genetic background for at least five filial generations (F5) before performing the anatomical and functional characterization. Mutant phenotype did not depend on gender^28^ and the animals investigated were not selected by sex.

### Antibodies

The following primary antibodies were used: mouse anti-acetylated-α-tubulin clone 6-11B-1 and mouse anti-FLAG clone M2 (Sigma-Aldrich Co. Ltd.); rabbit-anti-γ-tubulin, rabbit anti-calbindin and mouse anti-β actin clone AC-15 (Abcam Ltd.); mouse anti-activated β-catenin (anti-ABC, clone 8E7) and mouse anti-Ki67 (Merck Millipore Inc.); rabbit mAb anti-phospho-histone H3(Ser10) clone D2C8 and rabbit anti-β-catenin (Cell Signaling Technology Inc.); and rabbit anti-HOXB5 antibody (Insight Biotechnology). Mouse anti-PAX6 was deposited to the Developmental Studies Hybridoma Bank by A. Kawakami (DSHB Hybridoma Product PAX6). Secondary antibodies were Alexa-Fluor 350-, Alexa-Fluor 488-, Alexa-Fluor 568- and Alexa-Fluor 594-conjugated goat anti-mouse IgG and goat anti-rabbit IgG (Molecular Probes Inc.).

### Cell-line and *in vitro* cerebellar neuronal culture

The human neuroblastoma SHSY-5Y neuronal cell-line was obtained from the American Type Culture Collection (ATCC) and maintained in DMEM/Ham’s F12 medium supplemented with 10% foetal calf serum (FCS), under standard conditions (37 °C, 5% CO_2_). Cells were passaged at a split ratio of 1:10 twice a week. The derivation and culture of mouse embryonic fibroblasts (MEFs) has been described previously^30^. MEFs were grown in DMEM/Ham’s F12 supplemented with 10% foetal calf serum/1% penicillin streptomycin at 37 °C and 5% CO_2_. Mouse cerebellar granule neuron cells were isolated and cultured from P0 post-natal animals of both genotypes, essentially as described^31^. Dissociated cells were cultured in 24-well plates coated with 100 μg/ml poly-D-lysine at a density of 5 × 10^5^ cells per well. Purified mouse N-terminal Shh protein (R&D Systems Inc.) was used to stimulate cells at a final concentration of 1 μg/ml for 6 hr. Wnt3a-conditioned media was prepared as described previously^28^ and used to treat 1 × 10^6^ cells per well for 24 hr.

### Cloning, plasmid constructs and transfections

Plasmid pCMV1-FLAG-HOXB5 was obtained from Addgene, Cambridge, MA, USA (plasmid number 20983). For transfection with plasmids, cells at 80% confluency were transfected using Lipofectamine 2000 (ThermoFisher Scientific Inc.) according to the manufacturer’s instructions. For transfection with human siRNAs, we used validated Dharmacon ON-TARGETplus “SMARTpool” human siRNAs, as described previously^32^. Human siRNA target sequences are as follows: scrambled negative control 5′-UGGUUUACAUGUCGACUAA, 5′-UGGUUUACAUGUUGUGUGA, 5′-UGGUUUACAUGUUUUCUGA, 5′-UGGUUUACAUGUUUUCCUA; human *IFT88* 5′-AGUAAAGGUGAACGACUAA, 5′-AGGAAGUGCUAGCGGUGAU, 5′-AGGCAAAUGGAACGUGAAA, 5′-GAGAAUUAUAUGAUCGUGA; and human *TMEM67* 5′-CAUGAAUUCUUACGACUUU, 5′-GCAGUAAGUGGACGAGAAA, 5′-CCUUAAAAGAGAAGCGGAA, 5′-UGACUUAACUGCCGAAGGA.

### Immunofluorescence and confocal microscopy

Cells were seeded at 1.5 × 10^5^ cells/well on glass coverslips in six-well plates and fixed in ice-cold methanol (5 minutes at −20 °C) or 2% paraformaldehyde (20 minutes at room temperature). Permeabilization, blocking methods and immunofluorescence staining were essentially as described previously^28^. Primary antibodies were used at final dilutions of x200-1000. Secondary antibodies were diluted x500. Confocal images were obtained using a Nikon Eclipse TE2000-E system, controlled and processed by EZ-C1 3.50 (Nikon Inc.) software. Images were assembled using Adobe Illustrator CS4.

### Preparation of tissue sections, histology and immunohistochemistry

For whole mount images, mouse embryos were dissected and cerebella were washed thoroughly in 1 x PBS and photographed with a Zeiss Stemi 2000-C dissecting microscope. For tissue sections, mouse embryos or dissected tissues were fixed in fresh 4% (w/v) *para*-formaldehyde and embedded in paraffin wax. Thin sections (4μm) were cut onto “Superfrost Plus” slides (VWR International Ltd.) and deparaffinised and rehydrated by standard methods. Sections were stained with haematoxylin and eosin (BDH Chemicals Ltd.) for 2 min, then dehydrated in ethanol, cleared in xylene and mounted in DPX. For immunohistochemistry, epitope recovery was obtained by boiling in 1 mM EDTA pH8.0 for 2 min using a pressure cooker, followed by 30 min cooling. Blocking and application of primary antibodies was as described^28,32^. Appropriate HRP-conjugated secondary antibodies (Dako Inc.) were used (final dilutions of x10000-25000). Sections were developed in “Sigma Fast” 3,3′-diaminobenzidine (DAB) with CoCl_2_ enhancer and counterstained with Mayer’s haematoxylin (Sigma-Aldrich Co. Ltd.).

### Whole cell extract preparation and immunoblotting

Whole cell extracts (WCE) from confluent SHSY-5Y cells were prepared by standard methods. Ten μg WCE total soluble protein was analysed by SDS-PAGE (using 4–12% acrylamide gradient gels) and western blotting according to standard protocols using either rabbit polyclonal antisera (final dilutions of x200–1000) or mAbs (x1000–5000). Appropriate HRP-conjugated secondary antibodies (Dako Inc.) were used (final dilutions of x10000–25000) for detection by the enhanced chemiluminescence “Femto West” western blotting detection system (Pierce Inc). Levels of β-actin were used as a loading control for all immunoblotting experiments.

### Canonical Wnt activity (TOPFlash) luciferase assays

SHSY-5Y cells were co-transfected with 0.5 μg TOPFlash firefly luciferase construct (or FOPFlash, as a negative control); 0.5 μg of pCMV1-FLAG-HOXB5 expression construct, or empty pCMV-FLAG control); and 0.05 μg of pRL-TK internal control *Renilla* luciferase reporter construct (Promega Corp). Cells were treated with L cell control or Wnt3a-conditioned media, as described previously^28^. Luciferase activities were assayed with the Dual-Luciferase Reporter Assay system (Promega Corp.) on a Mithras LB940 (Berthold Technologies Inc.) luminometer. Raw readings were normalized with *Renilla* luciferase values, and the results reported are from three independent biological replicates.

### Quantitative real-time (qRT)-PCR

RNA was extracted from E12.5, E15.5 and P0 mouse embryos and newborn pups. Cerebral cortex and cerebellum were dissected from E12.5, E15.5 and P0 wild-type and *Tmem67*^−/−^ mouse embryos and newborn pups in cold PBS chilled on wet ice. Tissues were snap frozen and trimmed using a cryostat into 30μm thick sections. RNA was extracted from tissue using standard guanidinium thiocyanate and phenol chloroform extraction (TRIzol; ThermoFisher Scientific Inc). First strand cDNA synthesis was with a ThermoScript RT-PCR System (ThermoFisher Scientific Inc.), using conditions recommended by the manufacturer. Primer Express 3.0 software was used to design forward and reverse qRT-PCR primers with the following forward and reverse primer sequences: mouse *Actb*: 5′-TGTCCACCTTCCAGCAGATGT and 5′-GCTCAGTAACAGTCCGCCTAGAA; mouse *Shh* (exon 1): 5′-AAAGCTGACCCCTTTAGCCTA and 5′-TTCGGAGTTTCTTGTGATCTTC; mouse *Gli1* (exons 4/5 splice junction) 5′-TCGACCTGCAAACCGTAATCC and 5′-TCCTAAAGAAGGGCTCATGGTA; mouse *Ptch1* (exon2) 5′-GCTGTGGCTGAGAGCGAAGT and 5′-AAATATGAGGAGACCCACAACCAA; human *GAPDH* 5′-TACTAGCGGTTTTACGGGCG and 5′-TCGAACAGGAGGAGCAGAGAGCGA; human *IFT88* 5′-TGAAATAGCTTATGCGAGGTTTT and 5′-TATGTGAGCTGGTGTGGTGG; human *TMEM67* 5′-TTGAAAATATCGCTGGGCTC and 5′-TGAAACTCCATTTACGGGGA; human *HOXB5* 5′-TGTAAACTCCTTCTCGGGGCG and 5′-GTCTATTTCGGTGAAATTGG; human *AXIN2* 5′-CAAGGGCCAGGTCACCAA and 5′-CCCCCAACCCATCTTCGT; human *DKK1* 5′-CGGGAATTACTGCAAAAATGGA and 5′-GCACAGTCTGATGACCGGAGA. qRT-PCR reactions were performed essentially as described^28^ using SYBR Green PCR master mix (Applied Biosystem Inc.) and ROX reference dye (ThermoFisher Scientific Inc). Reactions were performed in duplicate for at least n = 3 independent biological replicates. PCR reactions were run on an ABI7500 real-time PCR machine (Applied Biosystems Inc.) using standard cycling conditions, with the ABI software used to analyse qRT-PCR with the standard curve method, normalized against the input amounts of mouse *Actb* or human *GAPDH*.

### RNA sequencing

Total RNA was extracted from tissue using TRIzol (ThermoFisher Scientific Inc). RNA samples were treated with a TURBO DNA-free Kit (Ambion Inc.) using conditions recommended by the manufacturers, and then cleaned with an RNA Clean & Concentrator-5 spin column (Zymo Research Corp). RNA was tested for quality and yield using a NanoDrop 1000 spectrophotometer and an Agilent 2100 Bioanalyzer. RNA was processed further only if samples were uncontaminated with phenol and the RNA Integrity Number (RIN) was >9.0. To minimize bubble PCR artefacts, we used 100 ng of purified total RNA in library preparation, following the “TruSeq” Illumina protocol. In brief, RNA was polyA-selected, chemically fragmented to about 200 nt in size, and cDNA synthesized using random hexamer primers. Each individual library received a unique Illumina barcode. RNA-seq was performed on an Illumina HiSeq2000 instrument with six libraries multiplexed per flow-cell lane using 100 bp single-end (n = 2 samples) or paired-end (n = 4 samples) reads. Single-end sequencing gave a minimum of 12.5 × 10^6^ reads (average 16.2 × 10^6^) whereas paired-end sequencing gave a minimum of 30.6 × 10^6^ reads (average 32.2 × 10^6^).

The quality of the reads were assessed using FastQC, and trimming of the bases with quality scores <20 was performed with Trim_Galore! (v 0.5.0). RNA sequence reads were mapped to the GRCm38 mouse reference genome using STAR aligner version 2.6.1b with the–outSAMtype BAM SortedByCoordinate option^33^. Gene-level counts were generated from the binary alignment map (BAM) files using the Python library HTSeq (v 0.9.0)^34^ and the *featureCounts* function^35^ in the Rsubread package (v 1.26.1) in R with Mus_musculus.GRCm38.94.gtf from the Ensembl database. A total of 54532 unique transcripts were detected across all six analysed samples. Count normalization and differential expression were performed using DESeq2^36^ with the application of the Benjamini-Hochberg false discovery rate (FDR) method to adjust the p values. Genes were called as differentially expressed (DE) if they passed a statistical cutoff of FDR p < 0.05 and if they contained an absolute log_2_fold-change (FC) > = 1. Principle component analysis (PCA) was performed and sample-to-samples distances were computed using the Euclidean distance. Figures, including the heatmaps and volcano plot were made in R with the following packages: ggplot2 (v 2.2.1), ggbeeswarm (v 0.6.0), pheatmap (v 1.0.8), and RColorBrewer (v 1.1.2). Differential usage of exons (DEU) from RNA-seq data was performed using the DEXSeq package (v 3.8) and visualized in R. RNA sequencing data is available from NCBI Sequence Read Archive http://www.ncbi.nlm.nih.gov/sra under study SRP048892 with accession numbers SAMN03106994, SAMN03106995, SAMN03106996, SAMN03106997, SAMN03106998 and SAMN03106999.

### Chromatin immunoprecipitation assays

Mouse cerebellar granule neuron cells were grown as described above, fixed in fresh 1% [w/v] formaldehyde and harvested. ChIP assays were performed using the EZ ChIP kit (Millipore Inc), using the standard conditions described in the manufacterer’s protocol. Anti-RNA Polymerase II (clone CTD4H8) was used a positive IP control (data not shown), and normal purified mouse IgG was the negative control. For qPCR analysis of the relative levels of mouse *Ctnnb1* promoter in input DNA and immunoprecipitated DNA, we used the following primers: 5′-GGGGAGAGAGAAGCAATGCA and 5′-GAAGAGGATATCACCCCGGT (promoter region −3982 to −3631 for RefSeq transcript NM_007614, mouse genome build mm10). An intragenic region in *Ctnnb1* intron 13 (primers 5′-GCTCTGAGTCTTCCAAGGGT and 5′-GCTCCCACATGACTCACTCT) was used as a negative control. The levels of bound material were normalized against input using the standard curve method.

### Statistical analyses

Normal distribution of data was confirmed using the Kolmogorov-Smirnov test (GraphPad Software). Pairwise comparisons were analysed with Student’s two-tailed t-test using InStat (GraphPad Software). Results reported are from at least three independent experiments. Error bars on bar graphs indicate s.e.m. The statistical significance of pairwise comparisons shown on bar graphs are indicated by: n.s. not significant, *p < 0.05, **p < 0.01, ***p < 0.001, and ****p < 0.0001. For cell populations, a minimum of 150 cells were counted from 10 separate fields of view.

## Results

### Cerebellar hypoplasia and morphological disturbances in Tmem67−/− embryos during late gestation

**Figure 1:**
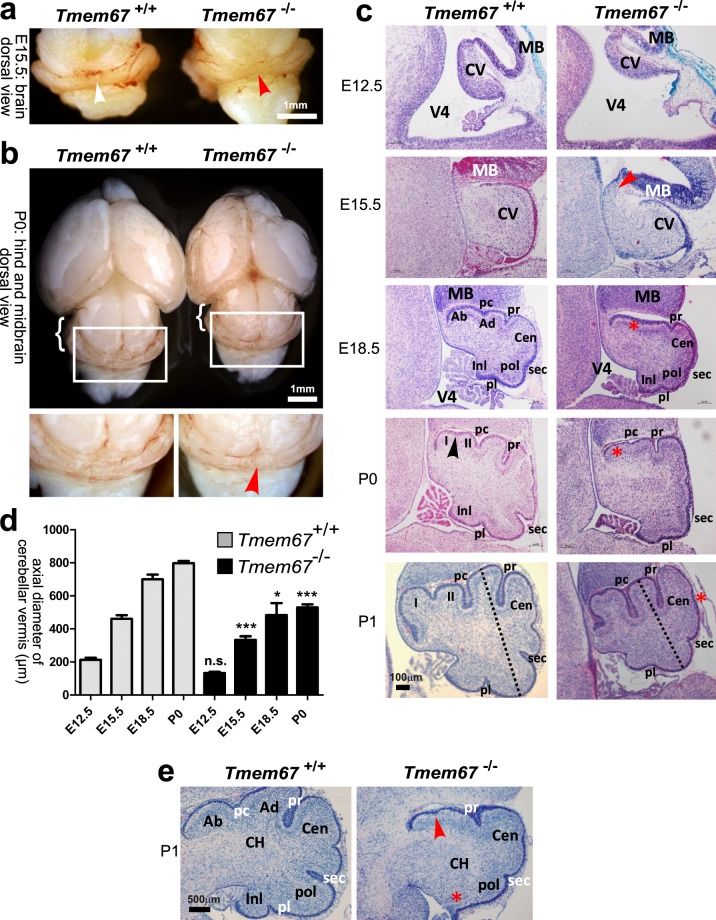
*Tmem67*^−/−^ cerebellar hypoplasia and foliation defects at perinatal and early postnatal stages of development. (**a**) Whole-mount E15.5 mid-hindbrain images showing dorsal views of the normal cerebellar vermis in the *Tmem67*^+/+^ embryo (white arrowhead) compared to the vermian hypoplasia/aplasia in the *Tmem67*^−/−^ mutant (red arrowhead). Scale bar = 1 mm. (**b**) Upper panel: dorsal view of whole mount P0 midbrain and hindbrain dorsal view of the whole brain and cerebellum at P0, with hemispheric hypoplasia indicated (bracket). Lower panels show magnified insets of the indicated boxes in the upper panel, showing vermian hypoplasia (red arrowhead). Scale bar = 1 mm. (**c**) H&E stained median sagittal brain sections showing vermian hypoplasia at the indicated developmental stages. The red arrowhead in the *Tmem67*^−/−^ E15.5 vermis denotes the thickened elongated superior cerebellar peduncle. At E18.5 note the foliation defects with delayed development (red asterisk) of the preculminate fissure (pc) in *Tmem67*^−/−^ animals. At P0 the non-principal fissure (arrowhead) in the anterobasal lobe developed, with formation of lobules I and II in *Tmem67*^+/+^, whereas the *Tmem67*^−/−^ mutant did not develop a principal fissure (red asterisk). At P1, the vermis was hypoplastic with delayed foliation, especially in the central lobe (Cen; red asterisk). The axial diameter between the antero-dorsal and postererior lobes of the vermis (dashed lines) is indicated for P1 sections. Scale bar = 100 μm in all panels. (**d**) Bar graphs quantitate the axial diameter between the antero-dorsal and postererior lobes of the vermis at the indicated ages. Statistical significance of pair-wise comparisons between age-matched animals is indicated by: n.s. not significant; *p < 0.05; ***p < 0.001; for Student’s t-test (paired, two-tailed). Error bars indicate s.e.m. (**e**) H&E stained parasagittal P1 cerebellar section, showing lack of preculminate (pc) fissure development (red arrowhead), absence (red asterisk) of the inferior lobe (Inl) and posterolateral fissure (Pl) in the *Tmem67*^−/−^ mutant. Scale bar = 500μm. Abbreviations: Ab, antero-basal lobe; Ad, antero-dorsal lobe; Cen, central lobe; CH, cerebellar hemisphere; CV, cerebellar vermis; Inl, inferior lobe; MB, midbrain; pc, preculminate fissure; pl, posterolateral fissure; pr, primary fissure; Pol, posterior lobe; sec, secondary fissure; V4, fourth ventricle.

Investigation of the cerebellum in *Tmem67*^−/−^ embryos at consecutive stages of embryonic development (E12.5, E15.5 and E18.5) and early postnatal stages (P0 and P1) showed consistent hypoplasia of the cerebellar vermis primordium (Fig. 1a,b), reduced axial diameter of the developing vermis **(**Fig. 1c,d**)** and reduced cerebellar size at all stages of development when compared to *Tmem67*^+/+^ wild-type littermates **(**Fig. 1c,d**)**. In mice, the developing cerebellar vermis of early to late mid-gestation embryos is normally a smooth structure with no foliation. The earliest indication of foliation starts to appear in the mouse cerebellum at E17.5 and at E18.5: the four principal fissures - the preculminate (Pc), primary (Pr), secondary (Sec) and posterolateral (Pl) - develop and divide the cerebellar vermis into five cardinal (antero-basal, antero-dorsal, central, posterior and inferior) lobes^37,38^. However, foliation during late gestation was defective in E18.5 *Tmem67*^−/−^ embryos and P0 pups (Fig. 1c). In mutants the preculminate (Pc) principal fissure failed to develop completely, whereas in *Tmem67*^+/+^ wild-type littermates the Pc fissure was fully developed and separated the anterior lobe primordium into antero-basal and antero-dorsal lobes. The other principal fissures were immature and poorly developed in *Tmem67*^−/−^ animals, whereas they were completely developed and demarcated the central, posterior and inferior lobes in wild-type littermates. By P1, the Pc fissure was developed in *Tmem67*^−/−^ animals whilst the other principal fissures remained shallower than in *Tmem67*^+/+^. Non-principal fissures remained immature throughout the gestation of *Tmem67*^−/−^ animals (Fig. 1c). Both cerebellar hemispheres were affected to the same extent as the vermis, and, frequently, with even more severe foliation defects with ectopic growth of the inferior lobe (Fig. 1e).

### Aberrant up-regulation of the canonical Wnt/β-catenin signalling in the *Tmem67*^−/−^ cerebellum during mid-gestation

**Figure 2:**
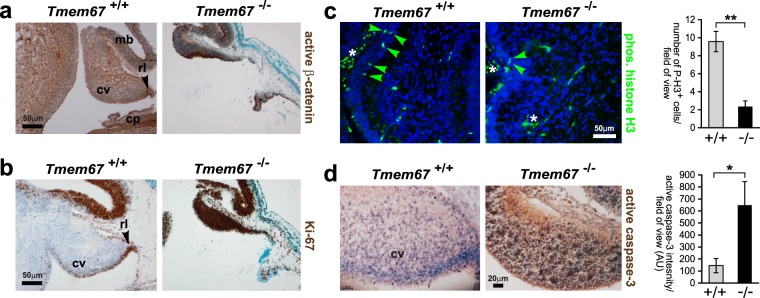
Over-proliferation and increased apoptosis at in the *Tmem67*^−/−^ cerebellum at E12.5. Mid-sagittal sections of the E12.5 cerebellar vermis stained for (**a**) active β-catenin; (**b**) proliferation (Ki67); (**c**) mitotic cells (P-H3, phospho-Ser10 histone H3; green, indicated by arrowheads), with bar graph on right to quantitate the number of P-H3^+^ cells per field of view for *Tmem67*^+/+^ (+/+) and *Tmem67*^−/−^ (^−/−^) cerebella; and (**d**) activated cleaved caspase-3, with bar graph on right to quantitate the intensity of IHC staining per field of view (AU; arbitrary units). In the wild-type embryonic cerebella, active β-catenin and Ki67 were largely restricted to the rhombic lip (arrowheads in **a** and **b**). Note the increase in canonical Wnt signalling (marked by active β-catenin), proliferation (Ki67) and apoptosis (active caspase-3), and the decrease in the number of mitotic cells (P-H3), in *Tmem67*^−/−^ sections. Asterisks indicate non-specific staining of red blood cells in (**c**). Scale bars = 50μm (**a**–**c**) and 20μm (**d**). Abbreviation: cp, choroid plexus; cv, cerebellar vermis; mb, mid-brain; rl, rhombic lip. Statistical significance of pairwise comparisons are indicated by: *p < 0.05; **p < 0.01; for Student**’**s t-test (paired, two-tailed). Error bars = s.e.m. for a minimum of 4 fields of view in n = 3 biological replicates.

Canonical Wnt/β-catenin-mediated signalling has an essential role during cerebellum development, with restricted spatio-temporal patterns of active Wnt/β-catenin signalling in the normal embryonic and perinatal developing mouse cerebellum^39^. We therefore investigated the status of Wnt/β-catenin signalling in the *Tmem67*^−/−^ cerebellum. The cerebellar vermis primordia of E12.5 *Tmem67*^−/−^ mutant animals were markedly hypoplastic or atrophic (Fig. 2a,b) to a variable degree in different mutant animals, as we have reported previously^28^. Immunohistochemical (IHC) staining showed an increase in the expression of both active β-catenin (Fig. 2a) and the proliferation marker Ki67 (Fig. 2b) in *Tmem67*^−/−^ mutant cerebellum. In the wild-type embryonic cerebellum, active β-catenin and Ki67 were largely restricted to the rhombic lip (Fig. 2a,b), as observed previously^39^. These results suggested that increased canonical Wnt/β-catenin activity, consistent with our previous findings in this animal model^28^, led to cellular over-proliferation in the *Tmem67*^−/−^ cerebellum at mid-gestation. Both the increased levels of active β-catenin and cell proliferation varied between different mutant animals which precluded reproducible quantitative analyses. However, these increases were concomitant with significantly decreased numbers of neuroepithelial cells labelled with the mitosis-specific marker phospho-histone H3 (Fig. 2c). Mutant vermis primordia also had significantly increased levels of apoptosis marked by active caspase-3 (Fig. 2d). The cerebellar hypoplasia and atrophy observed in the mutants at mid-gestation can therefore be ascribed to increased rates of proliferation and apoptosis, consistent with previous studies^39^.

### Loss of primary cilia in granule cell progenitors is concomitant with loss of responsiveness to Shh signalling in the developing *Tmem67*^−/−^ cerebellum

**Figure 3:**
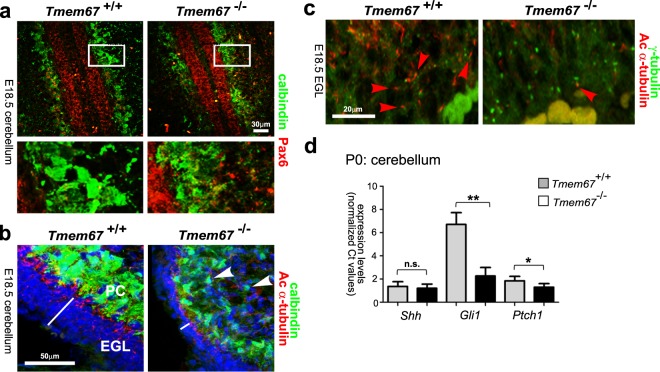
Defects in Purkinje cell morphology, ciliogenesis and Shh signalling in the *Tmem67*^−/−^ cerebellum. (**a**) E18.5 cerebellar tissue with GPC cells stained for Pax6 (red) and PCs stained for calbindin (green). Boxed areas are magnified in the bottom panels, showing details of the cellular architecture of the GPC and PCs. Scale bar = 30μm. (**b**) E18.5 cerebellar tissue sections for the indicated genotypes stained for calbindin (green), acetylated α-tubulin (red) and nuclei (DAPI; blue). White line indicates the thickness of the external granular layer (EGL). Arrowheads indicate smaller, irregular PCs in the *Tmem67*^−/−^ cerebellum. Scale bar = 50 μm. (**c**) Ciliogenesis defects in perinatal *Tmem67*^−/−^ cerebellum in tissue sections stained for acetylated α-tubulin (red; indicated by red arrowheads), γ-tubulin (green) and nuclei (DAPI; blue). Note the less frequent cilia in *Tmem67*^−/−^ and the many basal bodies with no apparent ciliary axoneme. Scale bar = 20 μm. (**d**) Relative expression levels of *Shh*, *Gli1* and *Ptch1* in *Tmem67*^−/−^ P0 cerebellum compared to *Tmem67*^+/+^ control littermate tissue. Values are the mean of at least n = 3 independent experiments, with qRT-PCR runs performed in duplicate. Statistical significance of pairwise comparisons are indicated by: n.s. not significant; *p < 0.05; **p < 0.01; for Student’s t-test (paired, two-tailed). Error bars = s.e.m.

For histological assessment of the cerebellum cytoarchitecture and layering in the P0 post-natal cerebellum, we stained for calbindin and Pax6, markers for the Purkinje cell (PC) and GCP layers, respectively. Mutant PCs appeared smaller, irregular and were not well-polarized into a distinct cell layer compared to the wild-type *Tmem67*^+/+^ PCs (Fig. 3a,b). This was accompanied by a reduction in the number of cell layers forming the EGL (Fig. 3a,b), although cell fate determination appeared to be otherwise intact in mutants. Staining for acetylated α-tubulin visualized primary cilia in PCs and GCPs of the EGL for both genotypes, but in the perinatal *Tmem67*^−/−^ cerebellum cilia were much less abundant (Fig. 3b). This was confirmed by staining cerebellar neurons for both acetylated α-tubulin and γ-tubulin to visualize the ciliary axoneme and basal bodies, respectively (Fig. 3c). This showed that some GCPs in the mutant cerebellum had short and rudimentary cilia, with the majority of basal bodies lacking an apparent ciliary axoneme. In *Tmem67*^+/+^ comparable sections, GCPs were ciliated with most of the basal bodies having a projecting ciliary axoneme (Fig. 3c).

Since Shh signalling is known to regulate the rapid expansion of the cerebellum in the perinatal stages^22,40,41^, we investigated expression levels of *Shh* and downstream targets. We used qRT-PCR to quantify levels of *Shh*, *Gli1* and *Ptch1* transcript levels in the wild-type and mutant cerebellum, normalized against expression of *Actb*. There were comparable levels of *Shh* expression in the early postnatal P0 cerebellum of *Tmem67*^−/−^ and wild-type littermates (Fig. 3d). However, the Shh downstream targets *Gli1* and *Ptch1* had statistically significant reductions of expression levels in the *Tmem67*^−/−^ cerebellum (Fig. 3d). These suggest that the lack of cilia in the mutant cerebellum results in defective responses to *Shh* secreted by the PCs at the early perinatal stages in *Tmem67*^−/−^ mutant animals.

### Ectopic up-regulation of Antp homeobox family gene expression in the post-natal *Tmem67*^−/−^ cerebellum

**Figure 4:**
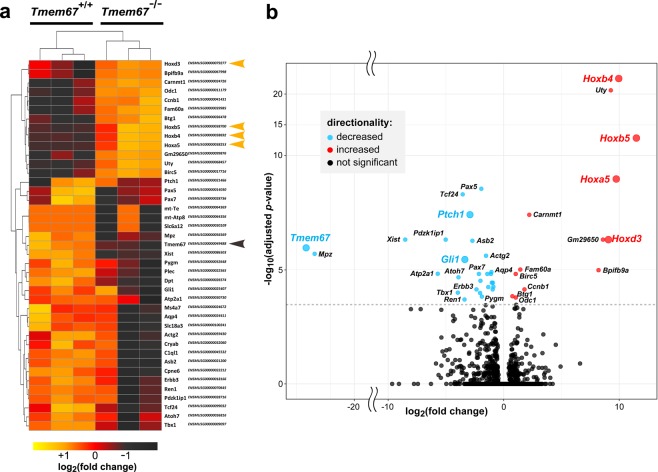
Differential expression analysis of the *Tmem67*^−/−^ post-natal cerebellum identifies a cluster of co-regulated homeobox transcription factors. (**a**) Heat map representing relative gene expression levels in early postnatal (P0) cerebella from matched littermate *Tmem67*^+/+^ *and Tmem67*^−/−^ animals (n = 3 biological replicates) for the indicated genes. All statisitically significant differentially expressed genes (total n = 41; Benjamini-Hochberg p-value adjusted for false discovery rate p_adj_* < *0.01) in all three data-sets are represented. Up-regulated genes in *Tmem67*^−/−^ mutants are coloured in dark-red/brown, with the co-regulated cluster of Hox genes indicated by orange arrowheads. Down-regulated genes are coloured in orange/yellow, with *Tmem67* indicated by the brown arrowhead. The DESeq2 output table for significant differentially expressed genes is shown in Supplementary Table S1. (**b**) Volcano plot displaying the 41 genes differentially-expressed in early postnatal *Tmem67*^+/+^ *and Tmem67*^−/−^ cerebella that pass statistical cut-offs of false discovery rate (FDR) p_adj_ < 0.05 and absolute log_2_fold change (FC) > = 1. The vertical axis (*y*-axis) corresponds to the statsticial significance of mean expression log_10_(p_adj_ value) and the horizontal axis (*x*-axis) displays the log_2_ fold-change values. Positive log_2_fold-changes (red points) indicate up-regulated expression in the *Tmem67*^−/−^ mutant compared to the *Tmem67*^+/+^ wild-type data-sets. Negative log_2_fold-changes (cyan points) indicate down-regulated expression in *Tmem67*^−/−^. Genes discussed in the main text are highlighted with coloured text and larger data points.

To delineate the gene regulatory and signalling pathways that were disrupted in the mutant cerebellum, we used RNA sequencing and analysis using DESeq2 to identify differentially expressed genes in early postnatal (P0) wild-type and mutant tissue. These analyses identified 41 genes (Fig. 4a,b) with statistically significant differential expression in three matched wild-type and mutant littermate pairs, following Benjamini-Hochberg adjustment for multiple testing to minimize the false discovery rates (p_adj_ < 0.01). The DESeq2 output table for significant differentially expressed genes is shown in Supplementary Table S1. *Tmem67* transcripts had an overall −24.121 log_2_ fold-change decrease in expression levels in the mutant cerebellum (p_adj_ = 0.00065; Supplementary Table S1). Statistically significant (p_adj_ < 0.01) decreases in diffferential expression (DE) and differential exon usage (DEU) for all exons or splice junctions in the canonical *Tmem67* transcripts were also observed (Supplementary Fig. S1). This data confirms that the *Tmem67*^*tm1*(*Dgen*)/*H*^ allele used in this mouse knock-out line is a true null allele. Furthermore, the RNA-seq data reiterated the results of our qPCR analysis (Fig. 3d), showing significant fold-change decreases for both *Gli1* (−3.361) and *Ptch1* (−2.954) in the *Tmem67*^−/−^ mutant cerebellum (Fig. 4b, Supplementary Table S1). As expected, there were no significant changes for either *Shh* (+0.340) or *Actb* (−0.066).

**Figure 5:**
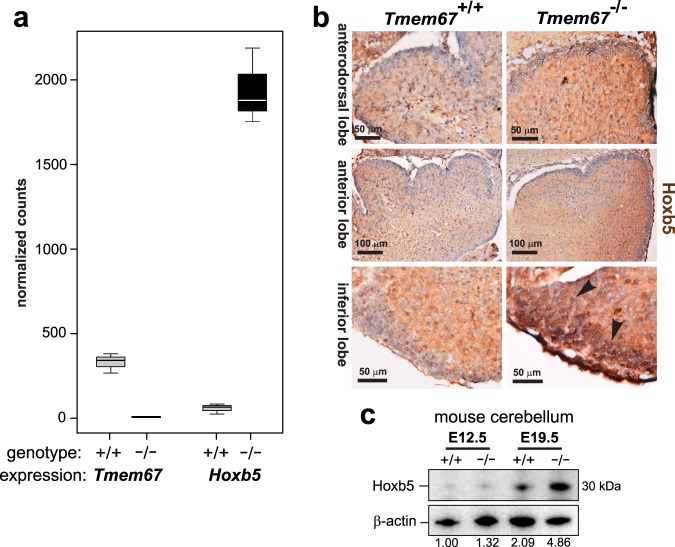
Homeobox transcription factor Hoxb5 is up-regulated in the *Tmem67*^−/−^ post-natal cerebellum. (**a**) Box plots of gene expression for *Tmem67* and *Hoxb5* gene expression in wild-type *Tmem67*^+/+^ (+/+) and mutant *Tmem67*^−/−^ (−/−) post-natal cerebellum RNA-seq datasets displayed as normalized counts of mapped reads. Whiskers show the minimum and maximum values, horizontal lines show the median and boxes indicate the 1st and 3rd quartiles. (**b**) Immunohistochemical staining for Hoxb5 in early postnatal (P0) cerebellum, showing increased protein expression in nuclei of mutant compared to wild-type lobes, especially for the inferior lobe (arrowheads). Scale bars are 50 or 100 μm, as indicated. **(c)** Western blotting of protein lysates from embryonic (E12.5) and perinatal (E19.5) *Tmem67*^+/+^ (+/+) and *Tmem67*^−/−^ (−/−) cerebellum for Hoxb5, with β-actin as a loading control. Relative protein level ratios for Hoxb5 and β-actin (loading control) are indicated. Full scans of blots are shown in the Supplementary Information File.

Hierarchical clustering analysis also revealed a single clustered, co-regulated group of homeobox-type transcription factors that were all significantly up-regulated in the mutant cerebellum (Fig. 4b). These included members of the Antp homeobox family (*Hoxb4 and Hoxb5*), and another member of the Hox paralogue group (PG) 5 (*Hoxa5*) (Fig. 4b). Further statistical analysis of gene expression for *Tmem67* and *Hoxb5*, the most significantly up-regulated Hox gene, confirmed loss of *Tmem67* expression and increased *Hoxb5* expression in the *Tmem67*^−/−^ mutant cerebellum (Fig. 5a). IHC staining for Hoxb5 confirmed increased protein expression in cell nuclei of early postnatal (P0) *Tmem67*^−/−^ mutant cerebellum compared to wild-type (Fig. 5b). Increased nuclear localization was especially pronounced in the inferior lobe of the mutant cerebellum (Fig. 5b). Western blotting for Hoxb5 in protein lysates also showed a significant increase in levels at E19.5 in *Tmem67*^−/−^ mutant cerebellum (Fig. 5c).

### *Ex vivo* cerebellar neuronal cultures implicate Hoxb5 in increased canonical Wnt/β-catenin signalling of *Tmem67*^−/−^ cerebella

**Figure 6:**
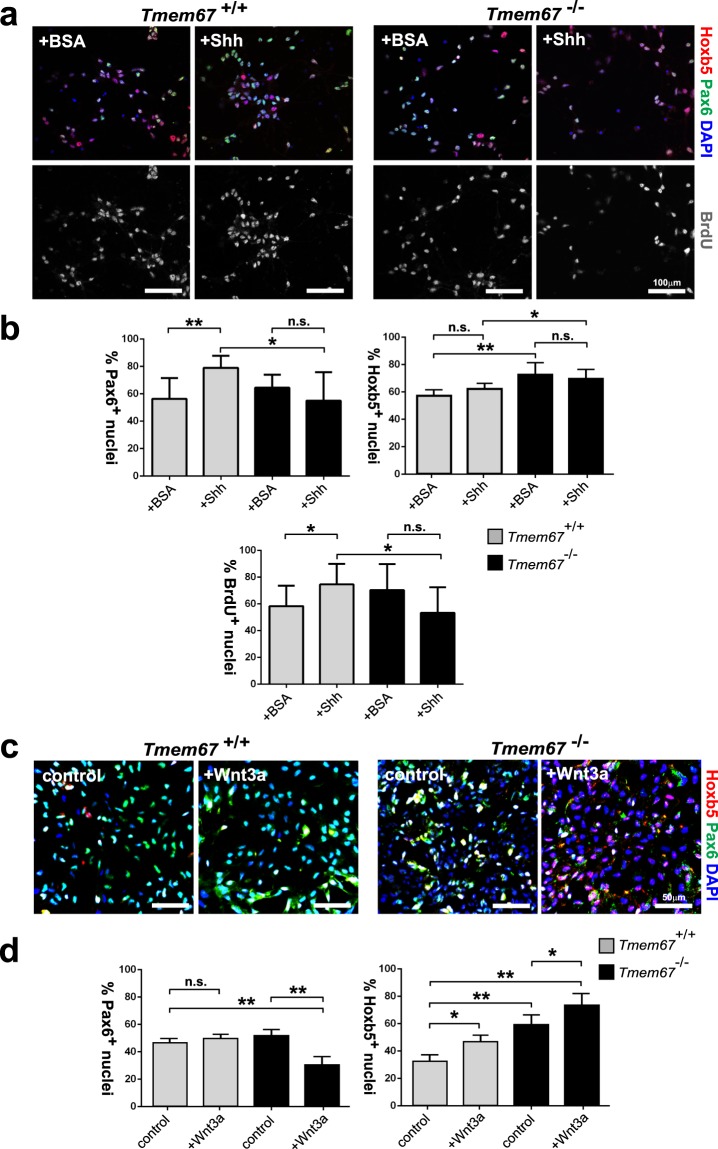
Wnt3a induces aberrant Hoxb5 expression and GCP loss in the early post-natal *Tmem67*^−/−^ cerebellum. (**a**) Representative images of *in vitro* cerebellar neuronal cultures from *Tmem67*^+/+^ and *Tmem67*^−/−^ early postnatal (P0) cerebellae stimulated with either exogenous, purified N-terminal Shh protein or negative control protein (BSA). Cells were stained for Hoxb5 (red), Pax6 to visualize GCPs (green), counterstained with DAPI (blue), and assayed for incorporation of BrdU (grey) to measure cell proliferation. Scale bars = 100μm. **(b)** Bar graphs to quantitate the percentages of Pax6^+^ nuclei (GCPs; top left panel), Hoxb5^+^ nuclei (top right panel) and BrdU^+^ nuclei undergoing proliferation (bottom panel) in cerebellar neuronal cultures for 10 fields of view in n = 3 independent experiments. (**c**) Representative images of *in vitro* cerebellar neuronal cultures from *Tmem67*^+/+^ and *Tmem67*^−/−^ early postnatal (P0) cerebellae stimulated with either L cell control or Wnt3a conditioned media, as for (**a**) above. Scale bars = 50μm. (**d**) Bar graphs to quantitate the percentages of Pax6^+^ nuclei (GCPs; left panel), and Hoxb5^+^ nuclei (right panel) in cerebellar neuronal cultures for 10 fields of view in n = 3 independent experiments. For all panels, statistical significance of pairwise comparisons are indicated by: n.s. not significant; *p < 0.05; **p < 0.01; for Student’s t-test (paired, two-tailed). Error bars = s.e.m.

To further investigate the role of Hox5b in cerebellar signalling, we used *ex vivo* cerebellar neuronal cultures stimulated with exogenous, purified N-terminal Shh protein or Wnt3a ligand and then stained for Pax6 to visualize GCPs and incorporated BrdU to measure cell proliferation (Fig. 6). Exogenous Shh stimulated both a significant increase of Pax6^+^ GCPs and of cell proliferation in *Tmem67*^+/+^ neurons but, as expected, these responses were absent in mutant *Tmem67*^−/−^ cells (Fig. 6a,b). Mutant *Tmem67*^−/−^ cerebellar neuronal cultures had a significant increase of Hoxb5^+^ mutant cells compared to wild-type cells, as expected from *in vivo* results, but treatment with purified N-terminal Shh protein had no further effect on Hoxb5 expression for either genotype (Fig. 6b). However, we observed a significant decrease in Pax6^+^ GCPs in mutant *Tmem67*^−/−^ cells following treatment with Wnt3a, as well as a significant increase in Hoxb5^+^ mutant cells (Fig. 6c,d). These results suggest that reduced number of GCPs in the *Tmem67*^−/−^ EGL and aberrant Hoxb5 levels are associated with the dysregulated canonical Wnt/β-catenin signalling observed in the ciliopathy disease state.

### Increased canonical Wnt signalling following loss of TMEM67 is dependent on HOXB5

**Figure 7:**
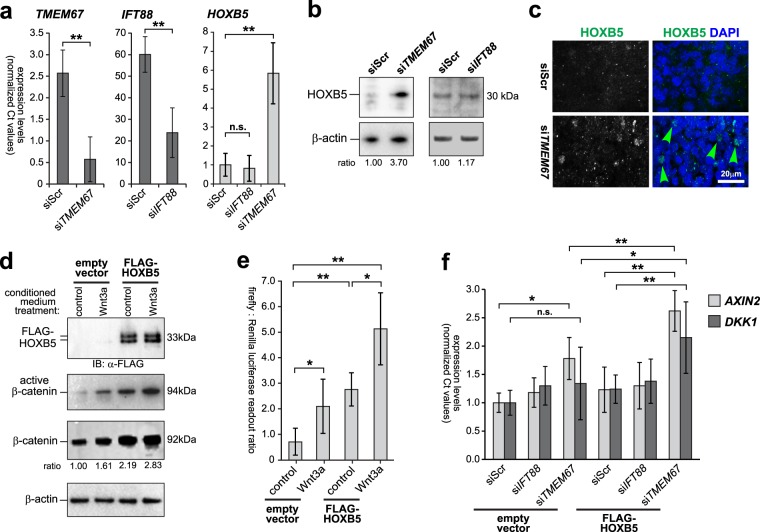
Increased canonical Wnt signalling following loss of TMEM67 is dependent on HOXB5. (**a**) Relative transcript expression levels of *TMEM67* (left panel), *IFT88* (middle panel) and *HOXB5* (right panel), following knockdown of either *TMEM67* (si*TMEM67*) or *IFT88* (si*IFT88*) compared to scrambled negative control (siScr). Statistical significance of pairwise comparisons are indicated by: n.s. not significant; **p < 0.01; for Student’s t-test (paired, two-tailed). Error bars = s.e.m. Values are the mean of n = 3 independent experiments, with qRT-PCR runs performed in duplicate. (**b**) Increased HOXB5 protein expression following siRNA knock-down of *TMEM67* but not *IFT88*. Relative protein level ratios for HOXB5 and β-actin (loading control) are indicated. (**c**) HOXB5 nuclear expression (green) is increased following *TMEM67* knockdown (green arrowheads). Scale bar = 20 μm. **(d**) Exogenous over-expression of FLAG-tagged HOXB5 (predicted size 33 kDa), visualized as a double band by immunoblotting (IB) with anti-FLAG (upper panel), increased expression of active β-catenin and total β-catenin. Treatment with Wnt3a conditioned media further increased active β-catenin and total β-catenin levels, compared to L cell control. Relative protein level ratios for HOXB5 and β-actin (loading control) are indicated. Full scans of blots are shown in the Supplementary Information File. (**e**) TOPFlash reporter assays showing increased canonical Wnt/β-catenin signalling following FLAG-HOXB5 over-expression and treatment with Wnt3a conditioned media, as indicated. (**f**) Activation of Wnt target genes *AXIN2* and *DKK1* was dependent on loss of TMEM67 and over-expression of HOXB5. For all panels, statistical significance of pairwise comparisons are indicated by: n.s. not significant; *p < 0.05; **p < 0.01; for Student’s t-test (paired, two-tailed). Error bars = s.e.m. Values are the mean of n = 3 independent experiments.

To provide functional evidence of a direct association between HOXB5 levels and canonical Wnt/β-catenin signalling, we used the human SHSY-5Y neuronal cell-line as an *in vitro* cellular model. As expected from the *in vivo* results, siRNA knock-down of *TMEM67* (Fig. 7a) caused marked increased expression of both the *HOXB5* transcript (Fig. 7a) and HOXB5 protein (Fig. 7b) that was localized in cell nuclei (Fig. 7c). However, siRNA knock-down of *IFT88* (Fig. 7a), encoding an essential component of ciliary IFT and ciliogenesis, had no effect on HOXB5 transcript or protein levels (Fig. 7a,b). This suggests that aberrant HOXB5 levels arise from a specific loss of TMEM67 rather than a general defect in ciliogenesis, and that the deregulation of canonical Wnt signalling caused by TMEM67 loss or mutation has a direct downstream effect on the gene regulatory pathways mediated by specific homeobox family genes including *HOXB5*.

To test this, we over-expressed FLAG-HOXB5 and activated canonical Wnt signalling by treatment with exogenous Wnt3a. HOXB5 over-expression and Wnt3a treatment increased both active β-catenin and total β-catenin protein levels (Fig. 7d). HOXB5 over-expression and Wnt3a treatment also had a significant additive effect on the functional activation of canonical Wnt/β-catenin signalling (Fig. 7e), as measured by the TOPFlash reporter assay. Knockdown of TMEM67 also led to the significant activation of the canonical Wnt target gene *AXIN2*, but not *DKK1* (Fig. 7f). However, there was a significant additive effect on the expression of both *AXIN2* and *DKK1* following TMEM67 knockdown and HOXB5 over-expression (Fig. 7f), suggesting that the increased canonical Wnt signalling following loss (or mutation) of TMEM67 is directly dependent on HOXB5. The result for *DKK1* in the cell-line was supported by an increased but non-significant change in expression for *Dkk1* (+2.005, p_adj_ = 0.101) in the *Tmem67*^−/−^ mutant cerebellum.

### Hoxb5 occupancy at the β-catenin promoter is increased by Wnt3a in *Tmem67*^−/−^ cerebellar neurones

**Figure 8:**
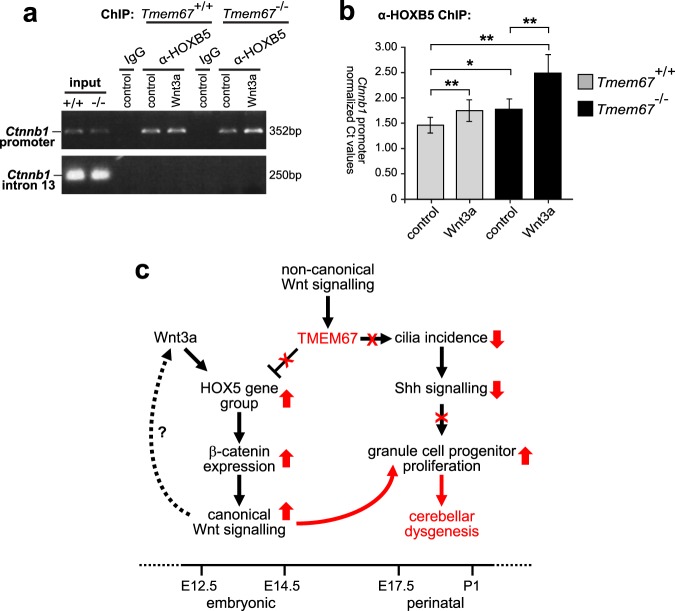
Hoxb5 occupancy at the β-catenin promoter is increased by activation of canonical Wnt signalling in *Tmem67*^−/−^ cerebellar neurones, informing the model of disrupted ciliary signalling in cerebellar hypoplasia. (**a**) Chromatin immunopreciptations (ChIP) using anti-Hoxb5 and control IgG for *in vitro* cerebellar neuronal cultures from *Tmem67*^+/+^ and *Tmem67*^−/−^ early postnatal (P0) cerebellae, following treatment with either control or Wnt3a conditioned media as indicated. Input and ChIP material was analysed by semi-quantative PCR for the β-catenin (*Ctnnb1*) promoter (top panel), and an intragenic region (intron 13) of the *Ctnnb1* gene as a negative control (bottom panel). Full scans of gels are shown in the Supplementary Information File. (**b**) Relative levels of the *Ctnnb1* promoter quantitated by qRT-PCR, for conditions as in (**a**). Values are the mean of at least n = 3 independent experiments, with qRT-PCR runs performed in duplicate. Statistical significance of pairwise comparisons are indicated by: *p < 0.05; **p < 0.01; for Student’s t-test (paired, two-tailed). Error bars = s.e.m. (**c**) Proposed model of disrupted signalling and gene regulatory pathways in the *Tmem67*^−/−^ mutant cerebellum (arrows indicate positive modulatory effects; blunt-ended arrows indicate repressive effects). Early in normal embryonic development (up to E14.5), active Wnt/β-catenin signalling is transient and restricted to either the cerebellar rhombic lip (time line indicated at the bottom). At the later perinatal period (E17.5 to P1) Shh stimulates granule cell proliferation, forming the EGL, in the absence of Wnt/β-catenin signalling. TMEM67 normally mediates non-canonical Wnt signalling to repress the expression of homeobox-type transcription factors, including the HOX5 gene group. Loss or mutation of TMEM67 (indicated by red text and symbols) releases the repression of HOX5 genes, causing increased β-catenin expression, dysregulated canonical Wnt/β-catenin signalling in early cerebellar development and aberrant proliferation of granule cell progenitors (upward-pointing red broad arrows). *Tmem67* mutation also decreases cilia incidence (downward-pointing red broad arrow) leading to loss of responsiveness to Shh signalling and further loss of correct modulation of neuronal proliferation. The timings of possible feedback loops in Wnt signalling (dashed arrow) remain unknown.

HOXB5 has recently been shown to bind to the β-catenin promoter and to regulate the expression of the human *CTNNB1* gene^42^, consistent with a transcriptional role in canonical Wnt/β-catenin signalling. We used chromatin immunoprecipitation (ChIP) to investigate if mouse Hoxb5 binds to one of the regions of the mouse *Ctnnb1* promoter (−3982 to −3631) that contains predicted putative HOX-binding TNAT motifs^42^. We confirmed that Hoxb5 does indeed bind to the mouse *Ctnnb1* promoter under normal conditions in *Tmem67*^+/+^ cerebellar neuronal cultures (Fig. 8a), but that this promoter occupancy was significantly increased following Wnt3a treatment in *Tmem67*^−/−^ cultures (Fig. 8a). As a negative control, Hoxb5 did not bind to an intragenic region (intron 13) of the *Ctnnb1* gene in which predicted HOX-binding motifs were absent (Fig. 8a). We then used qRT-PCR to quantify levels of the *Ctnnb1* (−3982 to −3631) promoter region after anti-HOXB5 ChIP from wild-type and mutant neurones, normalized against input chromatin material (Fig. 8b). ChIP analysis confimed the significant additive effect of exogenous Wnt3a and *Tmem67*^−/−^ genotype on HOXB5 occupancy of the *CTNNB1* promoter.

## Discussion

In early post-natal life, the mouse cerebellum undergoes over a thousand-fold increase in volume, largely due to the proliferation of the GCPs, which occurs simultaneously with the formation and growth of the folia^43^. Shh regulates the rapid expansion of the cerebellum in the perinatal stages, starting at E17.5 in mice, when it is secreted by PCs and diffuses to act as a mitogen to stimulate GCP cell cycle progression and proliferation^22,40,41^. Maturation of the PCs manifests as an increase in the size of the cell body, extension of the dendrites into the cell-sparse molecular layer and organization of the cells into a compact four- or five-cell layer during the very early postnatal stages, all of which coincide with the increase in post-natal cerebellar development and folia formation^37^. In the present study, we demonstrate that loss of the ciliary Frizzled-like receptor TMEM67, which is mutated in the ciliopathies MKS and JBTS, causes a severe cerebellar hypoplasia phenotype due to complex Wnt signalling, ciliogenesis and rostral hindbrain patterning defects that impact on downstream Shh signalling events. We observed that PC maturation appeared to be defective in the *Tmem67*^−/−^ cerebellum, since the PCs were smaller, irregular and did not polarize into a distinct cell layer (Fig. 3a,b). This is therefore likely to explain the pronounced foliation defects observed in the *Tmem67*^−/−^ cerebellum (Fig. 1c,d).

*Shh* expression correlates spatially and temporally with foliation^44,45^, and both *Shh* and its downstream effectors, the *Gli* proteins, are essential for GCP proliferation *in vivo*. Although in *Tmem67*^−/−^ mutant cerebella there was premature depletion of the GCP cell layer and PC immaturity (Fig. 3a,b), Shh expression was unaffected (Fig. 3d). However, GCPs had ciliary defects (Fig. 3c) and there were reduced expression levels of both *Gli1* and *Ptch1* in the *Tmem67*^−/−^ cerebellum (Fig. 3d). These observations suggest that GCPs have reduced responsiveness to Shh signalling, consistent with other ciliopathy mice models. The conditional *Kif3a* and *Ftm* mutant mice, for example, have cerebellar hypoplasia and foliation defects due to marked reduction in the Shh dependent expansion of GCP^46^. These observations have also been confirmed by the analysis of the cerebellum from *MKS3*/*TMEM67*-mutated human patients^29^. In addition, our observation of the selective delayed formation of the preculminate fissure **(**Fig. 1c**)**, that normally develops in the anterior cerebellar lobe, can also be attributed to down-regulation of the Shh target gene *Gli1* (Figs 3a and 4b) since it is selectively expressed in the anterior medial cerebellum^44^.

Our observation of increased Hox gene expression in the *Tmem67*^−/−^ cerebellum is unexpected because early developmental expression of, specifically, *Hoxa5* and *Hoxb5*, is normally required for the correct specification of rostral hindbrain cell-fates in rhombomere 1 ref.^47^. However, previous work has also shown specific expression of *Hoxb4*, *Hoxa5* and *Hoxb5* and *Hoxb7* in the early postnatal wild-type (P0) mouse cerebellum, with normal low levels of *Hoxb5* expression mainly in adult PCs^48^. Low level neo-expression of a group of Hox genes, including *Hoxa5*, were detected in the post-natal and adult cerebellum^49^, although *Hoxa5* expression appeared to be restricted to PCs and pericerebellar nuclei^50^. The latter constitute the main cerebellar afferent and give rise to mossy fibres that synapse with the cerebellar granule cells^51^. A postnatal wave of Hox gene expression then appears to be essential for the final development and maturation of PCs^52^. Recent work has indicated that HOXB5 is a direct transcriptional activator of β-catenin^42^, a finding recently confirmed in lung cancer cells^53^ and during lung alveoli development^54^. HOXB5 may therefore be an important transcriptional regulator of the canonical Wnt/β-catenin signalling pathway. Furthermore, consistent with a broader functional role for HOX5 group genes in Wnt signalling, mouse knock-outs of *Hoxa5* had reduced expression of the canonical Wnt ligands *Wnt2* and *Wnt7a*, as well as downstream targets of this pathway including *Lef1*, in developing lung tissue^55^. However, our data provides the first report of a functional association between Hox5 paralogue group genes and canonical Wnt signalling in the developing cerebellum. The observations of increased *Hoxb5* gene expression in the *Tmem67*^−/−^ cerebellum and increased occupancy of Hoxb5 at the β-catenin promoter (Fig. 8a,b) therefore provide a mechanistic explanation for increased canonical Wnt/β-catenin signalling in the ciliopathy disease state. This is supported by our findings that the activation of downstream Wnt target genes *AXIN2* and *DKK1* is also directly dependent on TMEM67 and HOXB5 (Fig. 7f). Interestingly, both of the Wnt target genes are also Wnt pathway components, indicating that feedback control of gene expression by HOXB5 may be a key feature of Wnt signalling regulation. Although these results were based on observations following knock-downs in the SHSY-5Y neuronal cell-line, the *Tmem67*^−/−^ mutant cerebellum also had evidence of disrupted Wnt signalling *in vivo* with increased (albeit non-significant) expression of *Dkk1*. This suggests that the cell-line is a relevant cellular model system of *in vivo* tissue, at least for some aspects of ciliary signalling.

Our data therefore suggest that the ciliary receptor TMEM67 is essential for both optimal levels of canonical Wnt/β-catenin signalling and the formation of primary cilia required for responsiveness to Shh signalling. We have recently shown that TMEM67 functionally interacts with the non-canonical Wnt signalling mediator Wnt5a, and that loss or mutation of TMEM67 prevents the normal modulation of the canonical Wnt pathway by non-canonical Wnt signalling through TMEM67^24^. To explain this observation, we suggest that TMEM67 mutation causes aberrant gene expression of Antp homeobox-type transcription factors that are normally associated with early rostral hindbrain patterning, including HOXB5, leading to increased β-catenin expression and increased Wnt/β-catenin signalling (Fig. 8c). In turn, this may signal aberrant proliferation of neuronal progenitors in the perinatal cerebellum, likely leading to apoptosis and cerebellar hypoplasia and dysgenesis (Fig. 2a–d). By contrast, in the normal developing embryonic cerebellum, high levels of active Wnt/β-catenin signalling are transient and restricted to the cerebellar rhombic lip (Fig. 2a,b). However, at the later perinatal period (E18.5 to P1), active Wnt/β-catenin signalling normally expands into a more widespread pattern with stronger expression at the ventricular zone but complete absence in the external granular layer^39^. These results are consistent with previous studies^56^ showing that over-expression of constitutively active β-catenin in the mouse cerebellar ventricular zone results in increased proliferation and impaired the capacity of cells for self-renewal and differentiation as early as E16.5. The severe cerebellar hypoplasia in the *Tmem67*^−/−^ knock-out model may therefore be due to the cumulative effect of aberrantly increased canonical Wnt/β-catenin signalling, originating earlier in embryogenic development, and later defective responses to Shh in the *Tmem67*^−/−^ cerebellum during perinatal development (Fig. 8c).

Previous work has shown that during post-natal cerebellar development, the non-canonical Wnt signalling pathway both antagonizes Shh signalling and suppresses GCP proliferation^57^. Since Wnt3a can also induce Hoxb5 expression^58^, confirmed by our data (Fig. 6c,d), we postulate that a positive feedback loop is unable to be modulated in the absence of TMEM67 (Fig. 8c). The loss of responsiveness to Shh signalling in the absence of cilia could indirectly remove possible modulatory effects on Hoxb5 expression and GCP proliferation, although recent work on a second Hox5 group gene, Hoxa5, has shown that it positively regulate *Shh* expression and its downstream target *Sm22a* in lung epithelium^55^. The HOX5 gene group may therefore mediate an additional positive feedback loop that regulates Shh signalling, at least in the developing lung. However, it remains unclear if the late defects we observe in the ciliopathy disease state arise from defective Shh signalling as a primary cause, or could also include an earlier loss of specification of rostral hindbrain patterning due to the disruption of Hox gene expression and canonical Wnt/β-catenin signalling. The mechanistic basis of the cross-talk between these complex gene regulatory pathways warrants further investigation, since it will provide further novel insights in the development and maturation of the mammalian cerebellum.

## Supplementary information

Supplementary Information
