# Supplementary material for: The ciliary Frizzled-like receptor Tmem67 regulates canonical Wnt/β-catenin signalling in the developing cerebellum via Hoxb5
Source: Sci Rep. 2019 Apr 1;9:5446. doi: 10.1038/s41598-019-41940-5 (PMC6445493; doi:10.1038/s41598-019-41940-5)
Supplement: Supplementary file 1 — Supplementary Information [file 41598_2019_41940_MOESM1_ESM.pdf]

The ciliary Frizzled-like receptor Tmem67 regulates canonical Wnt/β-catenin signalling in the developing cerebellum via Hoxb5

Zakia A. Abdelhamed, Dina I. Abdelmottaleb, Mohammed E. El-Asrag, Subaashini Natarajan, Gabrielle Wheway, Chris F. Inglehearn, Carmel Toomes, Colin A. Johnson

SUPPLEMENTARY INFORMATION

Supplementary Figure S1. Gene expression profile and differential exon usage plots for the *Tmem67* gene. RNA-seq data for wild-type *Tmem67*$^{+/+}$ and mutant *Tmem67*$^{-/-}$ post-natal cerebellum data-sets were analysed using DEXSeq (v 3.8). Expression and exon usage are shown for each exon or splice junction in the *Tmem67* gene (Ensembl gene ENSMUSG00000049488) from *Tmem67*$^{+/+}$ (blue traces) and *Tmem67*$^{-/-}$ (red traces) samples. Canonical Ensembl transcripts are shown beneath the main plotting panels, showing the location and organization of the corresponding numbered exons (E) in the gene. Genomic co-ordinates are indicated at the bottom. The predicted coding status of each transcript (protein coding, nonsense-mediated decay or retained intron) is also indicated. Differential expression (DE) and differential exon usage (DEU) was statistically significant ($p_{adj} < 0.01$) for all exons, with the greatest DE change identified for exon 41 encoding the 3'UTR (highlighted in magenta).

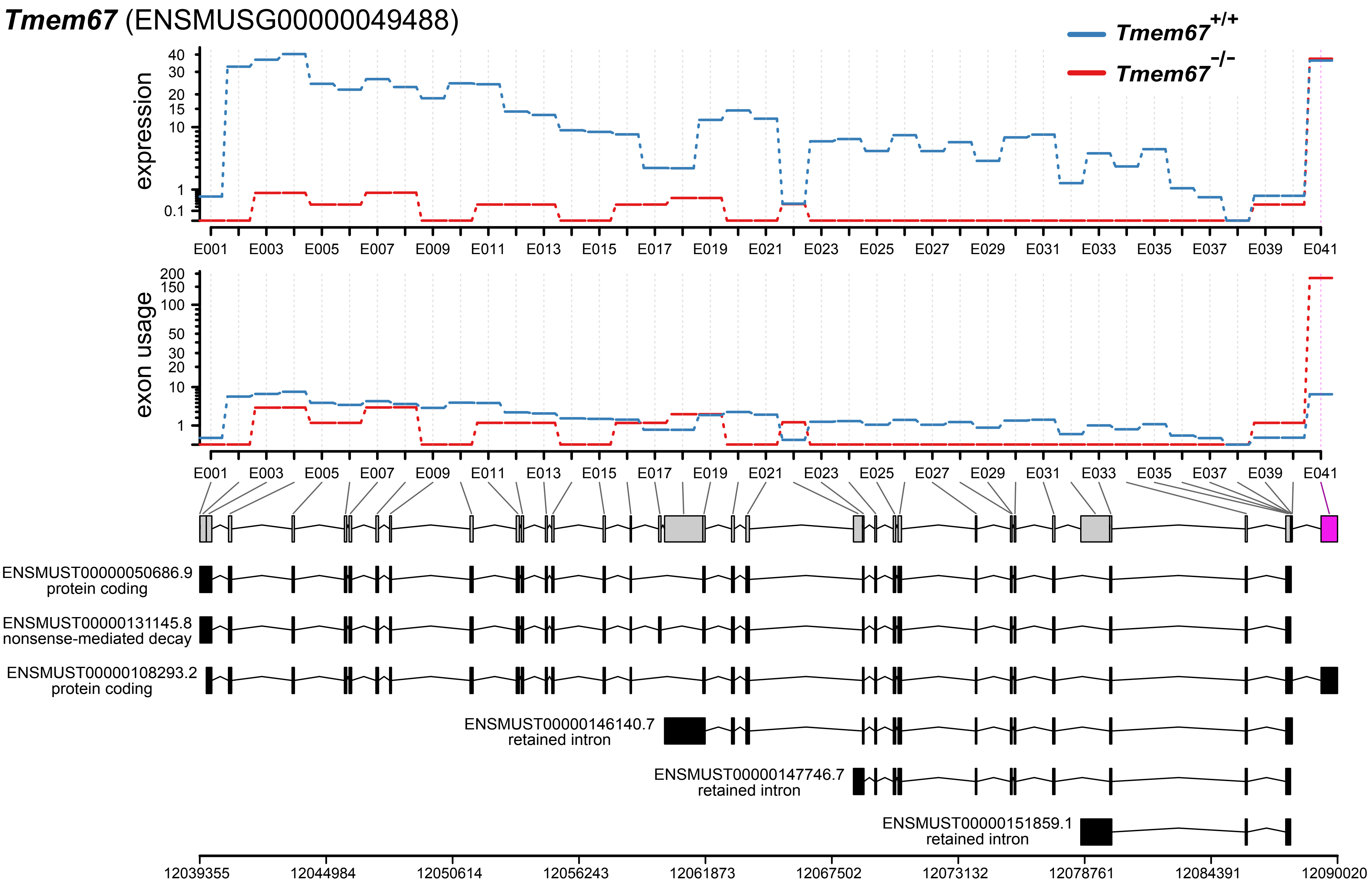

Tmem67 (ENSMUSG00000049488)
Tmem67+/+
Tmem67-/-
expression
40
30
20
15
10
1
0.1
exon usage
200
150
100
50
30
20
10
1
E001
E003
E005
E007
E009
E011
E013
E015
E017
E019
E021
E023
E025
E027
E029
E031
E033
E035
E037
E039
E041
ENSMUST00000050686.9
protein coding
ENSMUST00000131145.8
nonsense-mediated decay
ENSMUST00000108293.2
protein coding
ENSMUST00000146140.7
retained intron
ENSMUST00000147746.7
retained intron
ENSMUST00000151859.1
retained intron
12039355
12044984
12050614
12056243
12061873
12067502
12073132
12078761
12084391
12090020

Supplementary Table S1. DESeq2 output table for significant differentially expressed genes. RNA-seq data for the wild-type *Tmem67*$^{+/+}$ and mutant *Tmem67*$^{-/-}$ post-natal cerebellum data-sets were aligned using STAR and were analysed using DESeq2. In total, 41 differentially expressed (DE) genes passed statistical cut-offs of false discovery rate (FDR) $p<0.05$ and absolute $\log_2$fold change (FC) >=1. Column headings are as follows: baseMean, the average of the normalized counts taken over all samples; log2FoldChange, $\log_2$ fold change between *Tmem67*$^{+/+}$ and *Tmem67*$^{-/-}$ data-sets; lfcSE, standard error of the $\log_2$FoldChange estimate; stat, Wald statistic; pvalue, Wald test p-value; padj, Benjamini-Hochberg p-value adjusted for FDR. A positive $\log_2$ fold-change indicates that gene expression is up-regulated in the *Tmem67*$^{-/-}$ mutant compared to the *Tmem67*$^{+/+}$ wild-type, whereas a negative $\log_2$ fold-change indicates down-regulation.

Gene Symbol	ENSEMBL Gene IDs	baseMean	log2FoldChange	lfcSE	stat	pvalue	padj
Actg2	ENSMUSG00000059430	234.8231968	-1.529326491	0.315414687	-4.848621685	1.24322E-06	0.001440091
Aqp4	ENSMUSG00000024411	4202.190448	-1.065914248	0.235774735	-4.520901051	6.1577E-06	0.00577416
Asb2	ENSMUSG00000021200	55.68427508	-2.708290952	0.521846955	-5.189818444	2.10499E-07	0.000318858
Atoh7	ENSMUSG00000036816	479.4961287	-3.913345837	0.893244733	-4.381045521	1.18111E-05	0.008614243
Atp2a1	ENSMUSG00000030730	207.3356058	-5.707832431	1.273442549	-4.482206468	7.38752E-06	0.006612504
Birc5	ENSMUSG00000017716	480.2349764	1.049033209	0.263283461	3.984425019	6.76437E-05	0.033300999
Bpifb9a	ENSMUSG00000067998	32.07626809	8.209207121	1.79955039	4.561810087	5.07145E-06	0.004993351
Btg1	ENSMUSG00000036478	2547.518108	0.754291969	0.187886187	4.014621729	5.95412E-05	0.030854881
C1ql1	ENSMUSG00000045532	438.3182765	-0.954047577	0.230770315	-4.13418675	3.56E-05	0.020041603
Carnmt1	ENSMUSG00000024726	712.1717847	2.213849266	0.378720786	5.845597456	5.04753E-09	1.24245E-05
Ccnb1	ENSMUSG00000041431	783.8076346	1.799242084	0.433986056	4.145852291	3.38552E-05	0.020041603
Cpne6	ENSMUSG00000022212	394.7545703	-1.416058814	0.318413574	-4.447231308	8.69842E-06	0.006696636
Cryab	ENSMUSG00000032060	243.9493838	-2.042917525	0.502155696	-4.068295035	4.73584E-05	0.025204905
Dpt	ENSMUSG00000026574	132.746289	-2.010356229	0.465691003	-4.31693165	1.58213E-05	0.011126903
Erbb3	ENSMUSG00000018166	70.1724678	-2.355433048	0.569078316	-4.139031449	3.48775E-05	0.020041603
Fam60a	ENSMUSG00000039985	560.409657	1.438369814	0.313834092	4.583217211	4.57876E-06	0.004745523
Gli1	ENSMUSG00000025407	216.9142807	-3.360580954	0.704664748	-4.769049346	1.85097E-06	0.002024965
Gm29650	ENSMUSG00000099876	41.83200333	8.592388005	1.643491154	5.228131581	1.71232E-07	0.000280991
Hoxa5	ENSMUSG00000038253	541.1539434	9.270560642	0.887218392	10.44901766	1.48051E-25	1.45771E-21
Hoxb4	ENSMUSG00000038692	861.717211	9.9596331	0.89557464	11.12094141	9.92195E-29	1.95383E-24
Hoxb5	ENSMUSG00000038700	833.8199632	11.48531619	1.380966325	8.316869124	9.03174E-17	5.92843E-13
Hoxd3	ENSMUSG00000079277	57.987746	9.063517562	1.732736605	5.230753213	1.68821E-07	0.000280991
Mpz	ENSMUSG00000056569	78.66426876	-23.43263555	4.785588102	-4.896500714	9.75583E-07	0.001200699
Ms4a7	ENSMUSG00000024672	1449.406341	-1.107948178	0.249329151	-4.443716961	8.84179E-06	0.006696636
mt-Atp8	ENSMUSG00000064356	10383.90832	-0.937095054	0.21936786	-4.271797391	1.94E-05	0.01272784
mt-Te	ENSMUSG00000064369	126.3974416	-24.06295249	4.785270068	-5.028546382	4.94212E-07	0.000648801
Odc1	ENSMUSG00000011179	3128.060057	1.041450773	0.233502678	4.460123469	8.19124E-06	0.006696636
Pax5	ENSMUSG00000014030	291.6473746	-1.937064528	0.29390724	-6.590734292	4.37657E-11	1.72367E-07
Pax7	ENSMUSG00000028736	124.8429082	-2.145111601	0.48082438	-4.461320371	8.14562E-06	0.006696636
Pdzk1ip1	ENSMUSG00000028716	194.100041	-5.020535814	0.951229588	-5.277943283	1.30642E-07	0.000280991
Plec	ENSMUSG00000022565	2500.498594	-1.025714376	0.239663366	-4.279812946	1.87E-05	0.012701371
Ptch1	ENSMUSG00000021466	84.04040224	-2.954381119	0.505385159	-5.845801106	5.04136E-09	1.24245E-05
Pygm	ENSMUSG00000032648	332.2980605	-1.888237667	0.471682465	-4.003196656	6.24923E-05	0.031553811
Ren1	ENSMUSG00000070645	30.55767478	-3.389350606	0.85797731	-3.950396552	7.80218E-05	0.037473307
Slc18a3	ENSMUSG00000100241	246.6168674	-1.335045799	0.318604442	-4.190292488	2.78595E-05	0.017144046
Slc6a12	ENSMUSG00000030109	1009.704671	-0.893853081	0.212687342	-4.202662332	2.64E-05	0.016756872
Tbx1	ENSMUSG00000009097	27.15588889	-3.967570536	0.972601037	-4.079340229	4.51637E-05	0.024704542
Tcf24	ENSMUSG00000099032	91.01959095	-3.546393846	0.553302151	-6.409506703	1.45991E-10	4.79143E-07
Tmem67	ENSMUSG00000049488	130.5979608	-24.12131759	4.785253201	-5.040760976	4.63684E-07	0.000648801
Uty	ENSMUSG00000068457	250.1920761	9.742670022	1.411141753	6.904104426	5.05211E-12	2.48716E-08
Xist	ENSMUSG00000086503	11524.69877	-8.519539891	1.625710795	-5.240501518	1.60141E-07	0.000280991

Supplementary Information File. Full scans of gels and blots, with regions displayed in figures indicated by red frames.

Figure 4c

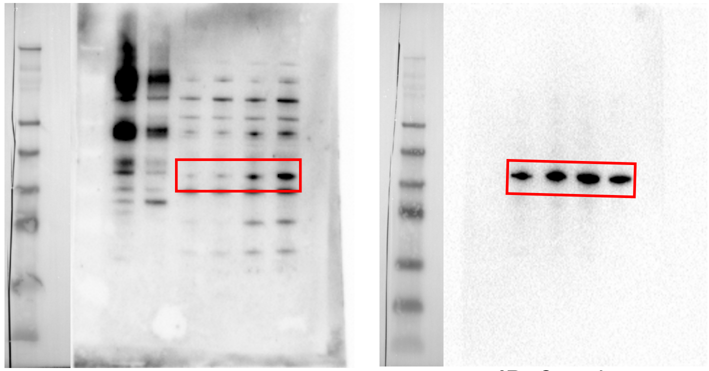

ladder IB: Hoxb5 ladder IB: β-actin

Figure 6c

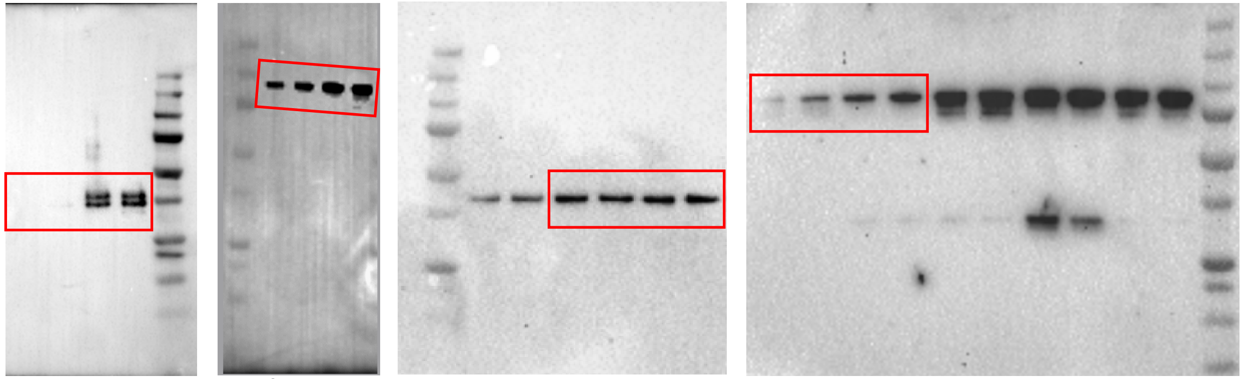

IB: α-FLAG IB: β-catenin IB: β-actin IB: active β-catenin

Figure 7a

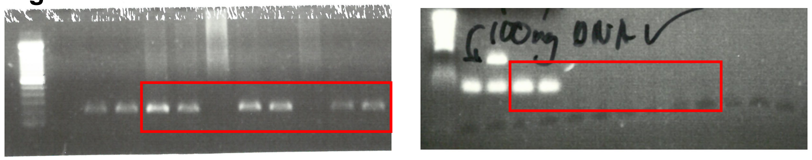

PCR: *Ctnnb1* promoter PCR: *Ctnnb1* intron 13
